# Méningite récurrente révélant une maladie de Behçet: à propos de deux cas

**DOI:** 10.11604/pamj.2014.19.14.1579

**Published:** 2014-09-08

**Authors:** Laila Lamzaf, Hicham Harmouche, Habiba Alaoui-Bennesser, Zoubida Tazi Mezalek, Mohamed Adnaoui, Mohamed Aouni

**Affiliations:** 1 Service de Médecine Interne, CHU Ibn Sina Rabat, Rabat, Maroc

**Keywords:** Méningite récurrente, maladie de Behçet, neuro-Behçet, Recurrent meningitis, Behçet disease, neuro-Behçet

## Abstract

Les étiologies des méningites sont surtout infectieuses. Les causes non infectieuses, sont souvent de diagnostic difficile et incertain, tel est le cas de la maladie de Behçet. Nous rapportons deux observations de patients ayant présenté des épisodes récurrents de méningite. Dans un premier temps, la suspicion d'une étiologie infectieuse a conduit à introduire un traitement anti-infectieux probabiliste. La découverte à posteriori d'une aphtose bipolaire a permis de poser le diagnostic de maladie de Behçet. Une corticothérapie s'est révélée efficace. La maladie de Behçet doit toujours faire partie des diagnostics différentiels des méningites récurrentes.

## Introduction

Les étiologies des méningites sont surtout infectieuses. Les causes non infectieuses, sont souvent de diagnostic difficile et incertain, tel est le cas de la maladie de Behçet. Nous rapportons deux cas de maladie de Behçet diagnostiquée au décours d'une méningite récurrente.

## Patient et observation

### Observation 1

Un patient de 25 ans présentant pendant un an des céphalées chroniques intermittentes, était hospitalisé pour un syndrome méningé fébrile remontant à une semaine. L'examen clinique notait une fièvre à 39.5°, une raideur de la nuque avec signes de Kerning et Brudzinski positifs. L'examen du fond d’œil était normal. L'analyse du liquide céphalorachidien (LCR) retrouvait 400 éléments/mm3 avec 97% de lymphocytes, une proteinorachie à 0.72 g/l et une glycorachie normale. L'examen direct, la culture ainsi que la recherche des antigènes solubles étaient négatifs. Le bilan biologique révélait un syndrome inflammatoire avec une vitesse de sédimentation (VS) à 48mm et une C réactive protéine (CRP) à 40 mg/l. La numération formule sanguine, les fonctions hépatique et rénale étaient normales. Le patient a été mis sous penicilline A à raison de 200 mg/kg/j pendant 10 jours avec bonne évolution; l'apyrexie a été obtenue à J2 du traitement, la ponction lombaire (PL) de contrôle retrouvait 4 éléments/mm^3^ avec un taux de protides à 0.23g/l. Un an plus tard, il était réadmis pour des convulsions fébriles avec troubles de conscience. L'examen clinique trouvait un patient fébrile à 39°, un Glasgow Coma Score à 10 sans signe de focalisation neurologique. Un scanner cérébral fait en urgence était normal. La PL avait ramené un liquide louche avec 2500 éléments/mm3 dont 90% de polynucléaires neutrophiles et une proteinorachie à 1.2g/l. L'examen direct, la culture et la recherche des antigènes solubles étaient négatifs. Au plan biologique, il existait un syndrome inflammatoire majeur (CRP: 120 mg/l - VS: 90mm) et une hyperleucocytose à 21000/mm^3^, à prédominance polynucléaires neutrophiles. La sérologie VIH était négative. Le malade a été mis sous penicilline A (200 mg/kg/j pendant 10 jours) avec bonne évolution clinique et biologique (PL de contrôle normale). Deux mois après, il a présenté pour la troisième fois un syndrome méningé. La PL retrouvait un liquide louche composé de 800 éléments/mm3 dont 60% de neutrophiles et 40% de lymphocytes, avec des protéines à 0.52g/l. L'examen direct, la culture et la recherche des antigènes solubles étaient négatifs. Le patient a été mis sous ceftriaxone 6 g/j pendant 10 jours avec évolution favorable (PL de contrôle normale). Un quatrième épisode de méningite est survenu un mois après, avec à la PL; 220 éléments/mm3 dont 90% de lymphocytes et une protéinorrachie à 0.84g/l. L’électrophorèse des protides dans le LCR avait révélé une hypergammaglobulinémie polyclonale modérée à 19.9g/l. La sérologie syphilitique aussi bien dans le sang que dans le LCR, la sérologie de Wright, de Lyme ainsi que la recherche de l'Herpes Simplex Virus par PCR étaient négatives. De même que la protéinurie de 24 heures et la recherche des anticorps antinucléaires. L'enzyme de conversion de l'angiotensine était normal. L’électrophorèse des protéines sériques avait objectivé une hypergammaglobulinémie polyclonale modérée à 17 g/l. Le patient a reçu de la ceftriaxone avec une évolution favorable (PL de contrôle sans anomalie). Pendant les 6 mois suivants, il n'a pas présenté de syndrome méningé mais il a rapporté 4 épisodes d'ulcérations orales et génitales. L'IRM cérébrale n'a pas objectivé d'anomalie. La recherche d'HLA B51 s'est révélée positive. Le diagnostic de maladie de Behcet était alors porté devant l'apparition de l'aphtose bipolaire. Le patient a reçu en conséquent de la prédnisone orale à la dose de 1 mg/kg/j avec dégression progressive associée à la colchicine 1 mg/j. Il est resté stable durant les 24 mois qui ont suivi.

### Observation 2

**Figure 1:**
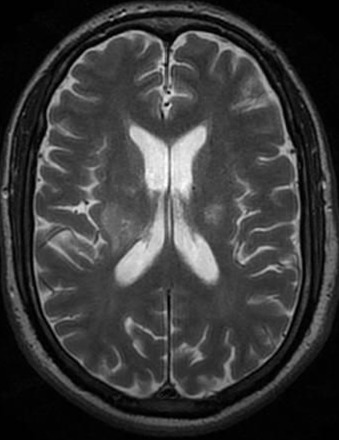
Coupe axiale en séquence T2: anomalie de signal de la substance blanche para-ventriculaire bilatérale à type d'hyper signal

**Figure 2:**
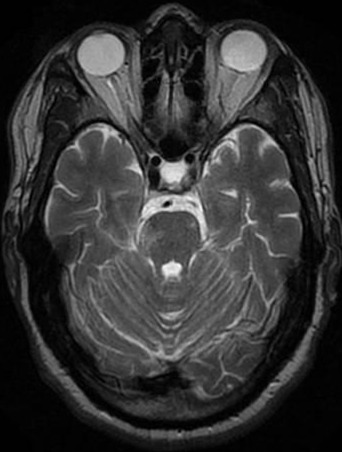
Coupe axiale en séquence T2: anomalie de signal latéroprotubérantiel droite à type d'hyper signal

**Figure 3:**
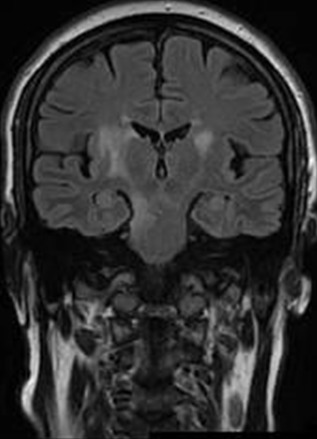
Coupe coronale en séquence FLAIR: anomalie de signal de la substance blanche para-ventriculaire bilatérale et latéro-protubérabtiel droite à type d'hyper signal

**Figure 4:**
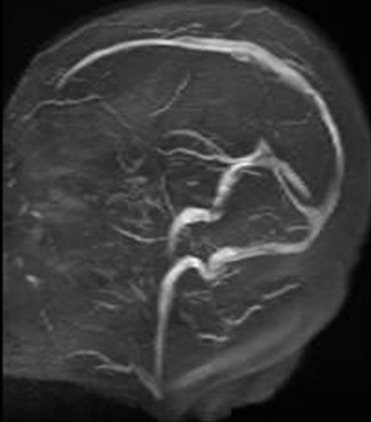
Séquence d'angio-IRM: bonne perméabilité des sinus veineux

Un patient de 52 ans, connu hypertendu depuis 5 ans sous régime seul, était admis pour un syndrome méningé fébrile. L'analyse du LCR retrouvait 650 éléments blancs/mm3 dont 82% de polynucléaires neutrophiles, une hyperprotéinorachie à 1,06 g/l et une glycorachie normale. L'examen direct, la culture ainsi que la recherche des antigènes solubles étaient négatifs. Le bilan biologique révélait un syndrome inflammatoire (VS à 50mm, CRP à 40mg/l). La numération formule sanguine, les fonctions hépatique et rénale étaient normales. Le patient a été mis sous penicilline A (200 mg/kg/j pendant 10 jours) avec bonne évolution; la PL de contrôle retrouvait 5 éléments/mm^3^ avec un taux de protides à 0.2g/l. Trois mois plus tard, il était réadmis pour des céphalées intenses, avec vomissements et hémiparésie gauche. L'examen clinique trouvait un patient apyrétique, normotendu. L'examen neurologique notait un ptosis de l’œil gauche et une hémiparésie gauche. On retrouvait également une aphtose buccale et génitale. L'IRM cérébrale mettait en évidence des anomalies bilatérales sous forme de plages en hyper signal T2 et Flair, avec absence de thrombophlébite en séquence angiographique (Figure 1, Figure 2, Figure 3, Figure 4). La PL retrouvait 312 éléments blancs, avec 84% de lymphocytes, un liquide normoglycorachique, normochlorurorachique et normoprotéinorachique. Le bilan biologique révélait une anémie normocytaire à 9g/dl, une hyperleucocytose à 12200/mm^3^ à prédominance polynucléaires neutrophiles et un syndrome inflammatoire (VS à 85mm et CRP à 191 mg/l). La fonction rénale était normale. Les anticorps antinucléaires étaient négatifs. La recherche d'HLA B51 est revenue positive. Le diagnostic de maladie de Behçet était alors posé. L'hypothèse d'un neuro-Behçet était retenue dans ce contexte. Le patient a reçu un bolus de méthyl-prédnisolone (15mg/kg/j pendant 3j) relayé par de la prédnisone orale à 1mg/kg/j avec dégression progressive et la colchicine 1mg/j. L’évolution était rapidement favorable sur le plan clinique et biologique (VS: 20mm; CRP: 4mg/l). Vu l'atteinte neurologique grave, un traitement immunosuppresseur à base de bolus de cyclophosphamide (15mg/kg/mois) a été associé. Le recul actuel est de 7 mois, le patient est à son 8ème bolus de cyclophosphamide, il est stable, sous 20mg/j de prédnisone.

## Discussion

Le diagnostic de la maladie de Behçet est essentiellement clinique. Il n'existe aucun signe biologique spécifique. L'International Study Group for Behçet's Disease (ISGBD) a proposé en 1990 une définition de la maladie basée sur des critères diagnostiques [1]. Cette définition a fait l'objet de critiques, car elle reflète mal certaines formes de début et ne prend pas en compte quelques aspects de la maladie, notamment les thromboses veineuses et l'atteinte neuroméningée. Nos deux observations illustrent bien ces critiques. Les manifestations neurologiques de la maladie de Behçet sont observées dans 20% des cas. Elles surviennent généralement dans la quatrième décennie et dans les dix ans suivant le premier symptôme.

En dehors des thrombophlébites cérébrales qui se traduisent majoritairement par des tableaux d'hypertension intracrânienne, deux mécanismes physiopathologiques sont retrouvés: l'atteinte parenchymateuse, liée à une atteinte inflammatoire des veines de petit et moyen calibre, ainsi que des méninges, le neuro-Behçet et l'atteinte des artères cérébrales, exceptionnelle, l'angio-Behçet [2–5]. Au cours du neuro-Behçet, le tableau clinique peut être très variable: troubles psychiques, céphalées, atteintes des nerfs crâniens, atteintes motrices centrales, troubles sensitifs, syndrome pyramidal, syndrome cérébelleux. L'angio-Behçet peut se traduire par un tableau d'accident vasculaire cérébral [2–5].

Dans la majorité des cas, l'atteinte méningée est latente ou de découverte fortuite lors d'une ponction lombaire systématique. Mais elle peut se présenter selon un mode aigu ou encore récurrent [6]. Ce dernier est exceptionnel [7–12]. Les observations que nous rapportons ont débuté par un tableau de méningite typique, le diagnostic de maladie de Behçet a été retenu après des épisodes récurrents de méningite et l'apparition de l'aphtose bipolaire. La méningoencéphalite, compliquant une maladie de Behcet, est d'installation aiguë ou subaiguë, la fièvre n'est pas constante, un tableau de rhombencéphalite est classique. L'atteinte du système nerveux est diffuse avec une prédilection pour le tronc cérébral et le diencéphale. L'imagerie cérébrale permet d'apprécier l'atteinte de la maladie de Behçet. Un scanner cérébral normal n’élimine pas la maladie, les images les plus fréquemment rencontrées sont des hypodensités multiples. L'IRM cérébrale est l'examen de référence pour le diagnostic et le suivi des lésions. Classiquement multiples, les lésions apparaissent sous forme d'hyper signal en séquences T2 et Flair. Elles touchent préférentiellement le tronc cérébral, les noyaux gris centraux et la substance blanche [13, 14]. Les lésions radiologiques sont fréquemment plus étendues que ne le laisse penser la clinique et sont réversibles sous traitement [3, 14]. Ces lésions ne sont pas spécifiques de la maladie de Behçet et peuvent se voir dans d'autres vascularites ou dans la sclérose en plaques.

Le 2^ème^ patient a présenté, en plus de l'atteinte méningée, une hémiparésie avec anomalies de signal à l'IRM, évoquant une méningoencéphalite. Une formule panachée du liquide céphalorachidien est classique. Cependant, comme nous l'avons constaté ici, une méningite purulente aseptique, ou lymphocytaire peuvent être observées [3, 5]. La concordance des signes cliniques, radiologiques et des divers examens biologiques réalisés chez nos patients, nous a fait traiter une étiologie très rare de méningite récurrente; la maladie de Behçet. Aucun protocole thérapeutique n'est consensuel [15]. La décision d'une corticothérapie d’épreuve est difficile. Mais elle mérite d’être tentée, en particulier devant les formes récurrentes, et après avoir éliminé les autres étiologies. Dans les deux cas, l’évolution était favorable sous corticothérapie. Un traitement immunosuppresseur a été associé chez le 2ème patient, devant l'atteinte neurologique grave, afin de diminuer le risque de rechutes.

## Conclusion

Une méningite peut constituer la présentation initiale d'une maladie de Behçet. Pour ce, l'atteinte neurologique au cours de cette affection mérite d’être mieux connue et mentionnée comme étiologie face à des tableaux de méningites récurrentes. Il est également important de la prendre en compte comme critère diagnostique additionnel de la maladie.
